# Accurate and easy method for systemin quantification and examining metabolic changes under different endogenous levels

**DOI:** 10.1186/s13007-018-0301-z

**Published:** 2018-04-26

**Authors:** Victoria Pastor, Paloma Sánchez-Bel, Jordi Gamir, María J. Pozo, Víctor Flors

**Affiliations:** 1 Associated Unit EEZ-UJI. Metabolic Integration and Cell Signaling Laboratory, Plant Physiology Section, Universitat Jaume I, Associated Unit to the CSIC, Castellón de la Plana, Castellón Spain; 2 Department of Soil Microbiology and Symbiotic Systems, Estacion Experimental del Zaidin (CSIC), Granada, Spain

## Abstract

**Background:**

Systemin has been extensively studied since it was discovered and is described as a peptidic hormone in tomato plants and other *Solanaceae*. Jasmonic acid and systemin are proposed to act through a positive feed-back loop with jasmonic acid, playing synergistic roles in response to both wounding and insect attack. Despite its biological relevance, most studies regarding the function of systemin in defence have been studied via *PROSYSTEMIN (PROSYS)* gene expression, which encodes the propeptide prosystemin that is later cleaved to systemin (SYS). Interestingly, hardly any studies have been based on quantification of the peptide.

**Results:**

In this study, a simple and accurate method for systemin quantification was developed to understand its impact on plant metabolism. The basal levels of systemin were found to be extremely low. To study the role of endogenous systemin on plant metabolism, systemin was quantified in a transgenic line overexpressing the *PROSYS* gene (*PS*+) and in a silenced antisense line (*PS*−). We evaluated the relevance of systemin in plant metabolism by analysing the metabolomic profiles of both lines compared to wildtype plants through untargeted metabolomic profiling. Compounds within the lignan biosynthesis and tyrosine metabolism pathways strongly accumulated in *PS*+ compared to wild-type plants and to plants from the *PS*− line. The exogenous treatments with SYS enhanced accumulation of lignans, which confirms the role of SYS in cell wall reinforcement. Unexpectedly, PS+ plants displayed wild-type levels of jasmonic acid (JA) but elevated accumulation of 12-oxo-phytodienoic acid (OPDA), suggesting that *PS*+ should not be used as an over-accumulator of JA in experimental setups.

**Conclusions:**

A simple method, requiring notably little sample manipulation to quantify the peptide SYS, is described. Previous studies were based on genetic changes. In our study, SYS accumulated at extremely low levels in wild-type tomato leaves, showed slightly higher levels in the PROSYSTEMIN-overexpressing plants and was absent in the silenced lines. These small changes have a significant impact on plant metabolism. SA and OPDA, but not JA, were higher in the PROSYS-overexpressing plants.

**Electronic supplementary material:**

The online version of this article (10.1186/s13007-018-0301-z) contains supplementary material, which is available to authorized users.

## Background

The welfare of consumers and the profits for farmers and producers through safe food production are increasing demands in society. These demands have led to a reduction in the use of chemical pesticides; alternative substitutions for pesticide includes activation of the plant immune system and the reinforcement of defences [1–3], competition for the ecological niches of pests and microbial pathogens by beneficial microbes [4], or stimulation of the plant for antimicrobial secreted molecules to combat potential threats [5].

Cell signalling for defence is regulated through secreted molecules that can be perceived by external membrane-localized receptors. The perception of these molecules generates extracellular inputs that trigger downstream signalling outputs, which can in turn modulate cellular functions. In plants, specialized receptors constantly track putatively dangerous signals, which can stimulate the immune response. This signalling initiates the cascade of defence reactions that can have a microbial origin, the so-called microbe-associated molecular pattern (MAMPs), or are generated in the host from damaged cells, known as the damage-associated molecular patterns (DAMPs). Both signals are present in the apoplast and are detected by the host via launch of Pathogen Associated Molecular Pattern-Triggered Immunity (PTI) [6]. An important group of DAMPs are secreted peptides, which function as key components of the immune system [5, 7, 8]. In fact, there is increasing interest in the role of peptides in development and defence processes [9]. For instance, several peptides produced by *Arabidopsis thaliana*, called AtPeps, and their receptors PEP-RECEPTOR 1 (PEPR1) and PEP-RECEPTOR 2 (PEPR2) have been recently described [10, 11]. Moreover, a homologue of AtPeps, ZmPep1, was discovered in *Zea mays* [12], and its active peptide homologues displayed activities in different plant species against biotic stress [13].

The peptide systemin (SYS) was categorized as a hormonal peptide and was the first described in its class. Interestingly, 25 years after the discovery of this peptide [14], its properties, mechanism of action and post-translational changes driving its biosynthesis are still under study. In a search for systemic signals that accumulate after a predator attack, Pearce et al. [14] found a peptide that could induce proteinase inhibitor (PI) activity when supplied to young tomato plants in a manner similar to wounding or application of oligosaccharides and methyl jasmonate [15–17]. This peptide was found in phloem and systemic tissues within minutes after wounding and thus was called “systemin.” SYS consists of a peptide sequence with 18 amino acids, which is generated by the proteolytic breakdown of the 200-amino acid protopeptide called PROSYSTEMIN [18]. Overexpression and antisense silencing of the *PROSYS* gene lead to constitutive expression or downregulation, respectively, of the plant defence gene *PI* [18, 19]. Therefore, the study of SYS-regulated mechanisms is of great interest, particularly because more peptides have been isolated and identified as modulators of plant defence. The PROSYS gene seems to act together with HypSys precursor genes for effective systemic signalling and amplification of the octadecanoid pathway [20], supporting the idea that peptides cleaved from their respective precursors are important players in defence response.

SYS is classified as a ribosomal peptide because of its mode of synthesis [5]. The levels and function of these peptides are difficult to predict through transcriptomic studies due to their small size and, in some cases, the lack of a specific site of cleavage. Many peptide receptors remain elusive [21]. Therefore, the use of alternative methods to detect and/or identify these small peptides is important. Liquid chromatography coupled to mass spectrometry can be efficiently employed for peptide analysis. The number of recent publications identifying peptides in complex biological samples is rising [22–24], although there are still no reports of reliable peptide quantification in a plant matrix.

In the present study, we aimed to quantitatively determine the plant hormone peptide SYS with a simple method and with little manipulation of the plant sample. We have developed a very sensitive method to determine SYS in complex plant matrices at very low concentrations. An over-expressed mutant and an antisense mutant of *PROSYSTEMIN* [18, 19] were used as positive and negative controls, respectively. Previous studies demonstrated that changes in *PROSYS* expression could be extrapolated to real levels of SYS in the plant; however, likely post-translational regulation has previously been ignored, and real levels of the peptide in different experimental conditions remains mostly unknown.

A simple plant extraction and chromatographic procedure to determine the peptidic hormones is provided. This knowledge may also be useful in quantifying other plant peptides. Furthermore, regarding the relevance of SYS as a plant regulator, metabolomic studies in the overexpressor and antisense lines were performed. This study aimed to decipher pathways that are either regulated or influenced by SYS. Variations in the endogenous levels of SYS provide insight into the role of this peptide. Interestingly, higher impact of SYS variation is found in the synthesis of lignans, which are important compounds for cell wall formation and fortification of plant-basal defences and biotic stress [25, 26].

## Results and discussion

### Optimization of an LC–MS/MS quantitative method for SYS determination

SYS effects in tomato plants have been studied since the early 1990s [14], although quantitative studies of this peptide in plant tissues remain few. In this paper, a reliable method for the quantification of this peptide in tomato plant samples is proposed.

**Fig. 1:**
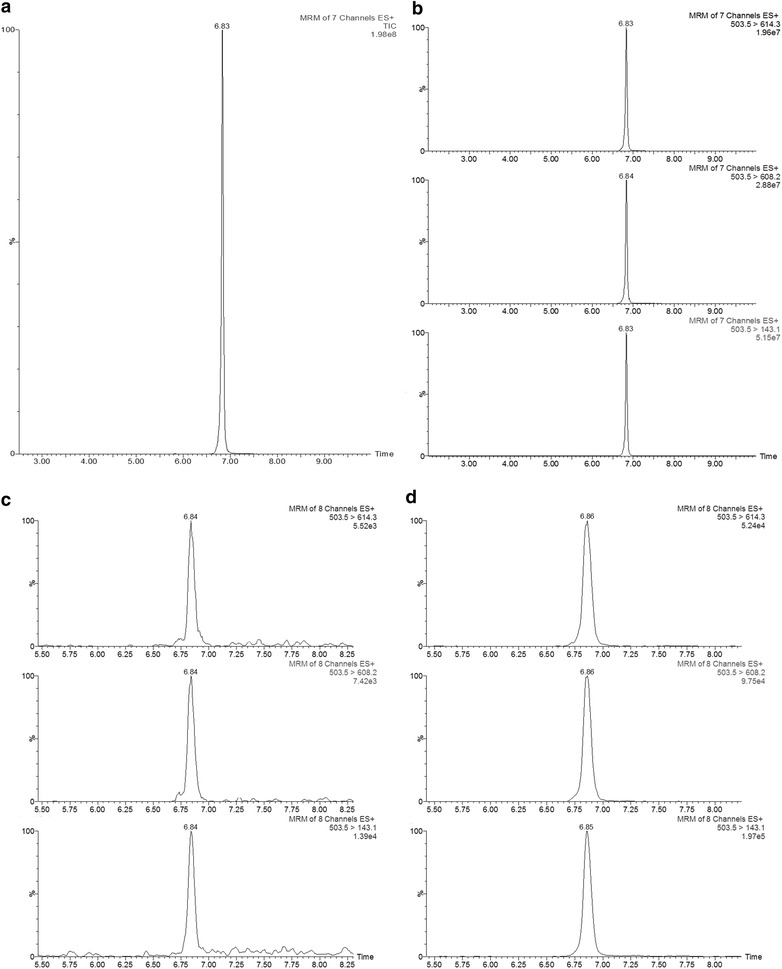
HPLC–MS/MS chromatograms for SYS in standard and plant samples. Main chromatograms obtained by 100 ng ml^−1^ of pure standard in H_2_O: ACN (9:1) v/v as both **a** total ion current of SYS in ESI (+) and **b** the three SRM transitions of the SYS standard. **c** SYS chromatogram for the three transitions obtained for SYS in the tomato plant sample and **d** the chromatogram for the same type of plant sample spiked with 150 ng ml^−1^

The Multiple Reaction Monitoring (MRM) procedure allows high specificity in the presence of complex sample matrices (such as plant extracts). This procedure can be achieved by selecting a specific precursor ion in the first quadrupole and then selecting a specific daughter fragment in the second quadrupole following fragmentation by the collision cell [27]. The total ion current of the SYS standard showed that the peptide eluted at 6.8 min (Fig. 1a), and the full scan MS spectrum of SYS showed the main signals at m/z 503.5 and 671.0 assigned to the [SYS + 4H]^4+^ and [SYS + 3H]^3+^ cations, respectively (Additional file 1: Fig. S1). Peptide precursor ion abundances were optimized by a full scan of SYS in H_2_O: ACN (9:1) v/v. The [SYS + 4H]^4+^ (m/z 503.5) cation was considered in the present study because of its higher signal response with respect to [SYS + 3H]^3+^ (m/z 671.0; Additional file 1: Fig. S1(a)).

MS/MS fragmentation of the [SYS + 4H]^4+^ (m/z 503.5) was optimized to reach maximum ion abundances of products, and this optimization displayed fragment ions at m/z 143.1, 608.2 and 614.3. Reasonably, the peaks obtained with these masses using the MRM detection elute at the same retention time provided an accurate tool for the identification of the peptide (Fig. 1b) and Additional file 1: Fig. S1(b)). To enhance reliability and accuracy of the method, the systemin heavy isotope [^13^C_5_, ^15^N]-SYS (SYS*) was used as an internal standard. This approach ensured identical chromatographic behaviour and m/z signal for the internal standard compared with the non-isotopic SYS. As the mass difference between the labelled and non-labelled SYS was more than two units in the parental ions and more than 5 units in some daughter ions, we avoided any possible overlapping due to natural isotopic abundance (Table 1b) [28]. For SYS*, the protonated [SYS* + 4H]^4+^ (m/z 505.0) was observed in its respective positive ESI mass spectrum, and the 505.0–148.1 m/z transition was optimized and selected for quantification purposes (Additional file 2: Fig. S2). The cone voltage and collision energy values for SYS and SYS* are summarized in Table 1a. As expected, both standards showed the same retention times (Additional file 3: Fig. S3).

**Table 1:** Optimized conditions for SYS quantification and method validation

(a)					
Compound	Weighted calibration equation	R^2^	Precision (%RSD) Intra-day/Inter-day	%RE	% Efficiency
Systemin	y = 1.11989x − 0.04709	0.9945	2.12/2.15	97.73	99.23

(b)				
Compound	Sequence	MRM (m/z)	Cone voltage (V)	Collision energy (eV)
SYSTEMIN	AVQSKPPSKRDPPKMQTD	503.5 > 143.1	35.0	14.0
		503.5 > 608.2	35.0	14.0
		503.5 > 614.3	35.0	14.0
SYSTEMIN*	A{Val(13C5, 15 N)} AVQSKPPSKRDPPKMQTD	505.0 > 148.1	35.0	14.0

(a) Parameters of the calibration curve for SYS and parameters used for method validation. *RSD* relative standard deviation; *RE* recovery. LOD (0.011 µg ml^−1^) and LOQ (0.033 µg ml^−1^) was calculated by signal to ratio noise of 3 and 10 respectively. A weighting factor of 1/x^2^ was applied to the curve

(b) Optimized MS/MS conditions for the analysis of SYS. Optimal conditions were selected by infusing the standards of SYS and SYS* to determinate appropriate cone and collision energies to obtain the characteristic transitions for each peptide

To quantify the peptide SYS, an HPLC–MS/MS method was validated regarding selectivity, linearity, precision, limit of detection (LOD) and quantification (LOQ), recovery and efficiency (Table 1a). LOD and LOQ has been evaluated based on building a calibration curve on the plant matrix of *Ps*− since the silenced plant lack or has not detectable levels of SYS. LOD has been calculated as 3.3 × (SDy/slope) and LOQ by multiplying the same factor per 10. The process efficiency has been determined by comparing plant material samples of BB (n = 6) spiked with the labelled standard before the extraction with a solution of pure labelled standard with a final concentration of 100 ng ml^−1^. This ratio is expressed in percentage. To improve peak resolution, as shown in Fig. 1c, SYS was detected in natural samples using all three selected transitions. Moreover, the transition corresponding to the internal standard was analysed in plant samples (BB, PS+ and Ps−) and there was no mass interference detected.

**Fig. 2:**
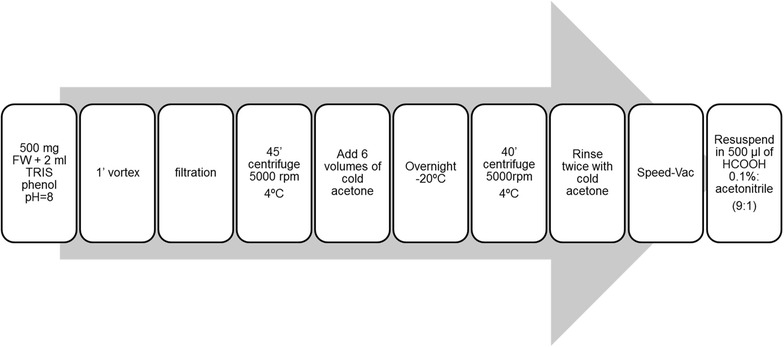
Graphical workflow for the analytical procedure. Steps to follow for an easy extraction of plant peptides and posterior injection in HPLC-MS/MS. The final residue was re-suspended as mentioned in the figure and injected into the TQD-MS/MS

Moreover, the chromatographic retention and identity were confirmed by fortification of the natural sample with the commercial standard of SYS (Fig. 1d) in a concentration of 150 ng ml^−1^. Thus, the basal levels of SYS in the tomato plants could be quantified with very good sensitivity and accuracy. Among the many experiments performed to quantify SYS, and due to the natural variability of SYS, we observed variability in basal SYS levels, detecting very low levels in certain experiments, but they may fall below LOQ in others. Nevertheless, the sensitivity in our experiments was good enough for detecting small changes in the SYS concentration in response to environmental changes. Notably, we first analysed SYS using an Acquity TQD (www.waters.com) instrument, although it was not sensitive enough to even detect the standards at high concentrations in our experimental conditions. Accurate SYS quantification was only possible using a Xevo TQ-S instrument (www.waters.com). The extraction of SYS from tomato leaves was performed as shown in Fig. 2: 500 mg of fresh material, stored at − 80 °C, was homogenized in a tube with 2 ml of Phenol/TRIS and saturated (ACROS Organic, ref. 327125000) at pH = 8. The mixed suspension was filtered by hydrophilic PVDF filter with a 25-mm diameter and a pore size of 0.45 µm (FILTER-LAB). After centrifugation, 6 volumes of pure cold acetone (Scharlau, AC0312, Pharm pur^®^) were added to each sample, and samples were kept overnight at in − 20 °C. The precipitate was recovered the next day and rinsed twice with cold acetone. The liquid phase was discarded, and the pellet was dried. The final residue was re-suspended in 500 µl of a solution of H_2_O (HCOOH 0.1%): acetonitrile (9:1, v/v) and injected into the TQS-MS/MS instrument.

Some tentative methods have been previously proposed for SYS detection [29, 30]. These methods are suitable for detection and quantification, although the manipulation of the sample is sensitive to possible degradation of the plant material. The QQQ (TQS) mass analyser used in our work was more suitable for quantification than the QTOF, with the latter being more accurate for exact mass measurement. Our method has two advantages. First, the handling proposed in the present method is simpler with no digestion step. By avoiding the digestion step, the co-elution of peptides derived from the digestion reduced the signal-to-noise ratio and increased the background signal, thereby improving the sensitivity of the method. Second, the use of a labelled internal standard ensured accuracy and precision, moderating the matrix effect due to the complexity of the plant sample and avoiding losses during extraction [24].

### SYS levels in tomato *PROSYSTEMIN* antisense and overexpressor lines

**Fig. 3:**
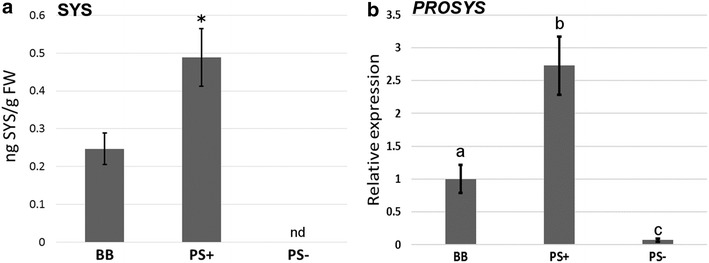
Quantification of SYS and expression of the *PROSYS* gene in tomato leaves. Levels of **a** SYS peptide and **b** *PROSYS* gene expression in leaves of tomato plants in wild-type plants (BB), the overexpressor of PROSYS (PS+) and the antisense of PROSYS (PS−). Data represent the mean and standard deviation (n = 6). The experiment was repeated three times, and asterisks indicate significant differences as determined by Student’s t-test, α = 0.05. No detection represented by nd. Different letters indicate statistically significant differences (ANOVA, Fisher’s least significant difference (LSD) test, *p *> 0.05, n = 6)

Pearce and colleagues described the presence of SYS in plants for the first time [14]. Surprisingly, an accurate method for SYS quantification is still not available, probably due to low basal levels of SYS in plants and the lack of appropriately sensitive instrumentation. *PROSYS* expression and/or responsive mutants were employed in this research to study the signals underpinning possible SYS effects in plants, such as its function as plant defence activator at the local or systemic level [31–33]. Several studies were performed using transgenic lines, which overexpressed or blocked the expression of *PROSYS* [33, 34]. Nevertheless, accurate quantification of shifts in SYS levels is still missing. In the present study, basal levels of SYS were determined using the wild-type plants of tomato *BetterBoy* (BB) cultivar and transgenic *35S::PROSYS* (PS+) and antisense PROSYS (PS−) lines [18, 19]. The basal levels of SYS could be quantified in the range of ng.g^−1^ of fresh weight (FW). The levels of SYS in the overexpressor line were twice the amounts found in wild-type plants and remained under the detection limits in PS− antisense plants (Fig. 3a). These results confirm the low *in planta* levels of this peptide, even in the overexpressing PS+ plants. McGurl and colleagues [19] showed a constitutive higher expression of *PROSYS* in transgenic plants than in the control, together with a systemic enhancement of the *PI1 and PI2* genes. Interestingly, the basal levels detected in wild-type plants were not enough to induce these systemic responses. Therefore, they proposed that PROSYS synthesis and subsequent processing to deliver SYS must be a regular process, but that SYS might remain compartmentalized until stress appears in wild-type plants. Thus, for overexpression, SYS delivered in the phloem may saturate the process, producing a continuous release of the peptide. However, SYS circulation may increase in wild-type plants only after wounding, inducing the transcription of proteinase inhibitors. As expected, the profile of SYS levels with respect to the expression levels of the *PROSYS* gene correlates (Fig. 3b). The basal amount of SYS found in PS+ plants was not as high as expected for an over-expressing line. This finding may suggest a strong post-translational regulation of the precursor’s cleavage and transport to phloem, supporting the relevance for the development of simple methods for peptide quantification. A recent publication provides relevant information about the mechanisms regulating the SYS levels by PROSYS processing: Beloshistov and colleagues [35] identified two genes, *Sl12g088760* and *Sl04g078740*, which encode for two phytaspases, named *Sl*Phyt-1 and *Sl*Phyt-2, respectively. These enzymes are aspartate-specific proteinases, which can hydrolyse the two aspartate residues flanking the SYS sequence in the precursor PROSYS leading to the cleavage of PROSYS into SYS. Undoubtedly, the evolution of this field is highly promising, particularly because the development of new analytical techniques can be suitable for the identification of post-translationally modified proteins [27, 36].

### SYS has a major impact in the plant metabolic profile

**Fig. 4:**
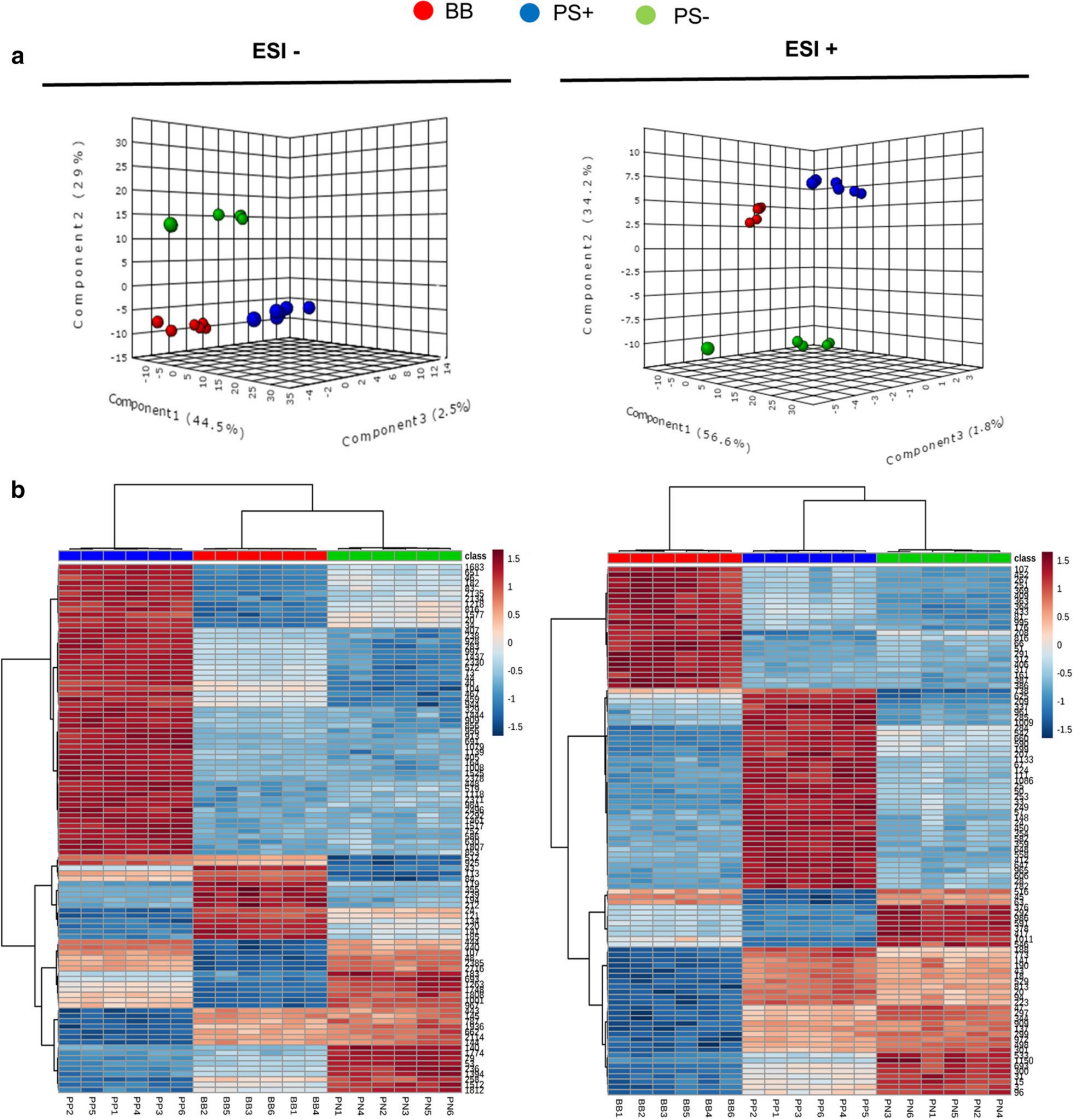
General outlook of the metabolic behaviour of the three genotypes of tomato plants using partial least squares, heatmap and clustering analysis. **a** 3D partial least squares plot explaining the major sources of variability for ESI (−) and ESI (+) signals obtained from a non-targeted analysis by UHPLC-QTOF MS in the three conditions BB, PS+ and PS−. Data points represent replicates of three independent experiments with two technical replicates per experiment and condition, which were injected randomly in the UHPLC-QTOF MS. Signals corresponding to different conditions were compared using the non-parametric Kruskal–Wallis test, and only data corresponding to *p *< 0.05 between groups were used for supervised analysis. **b** Heat map analysis and clustering of the different main signals corresponding to metabolite profiling, and generated with the Metaboanalyst 3.0 software, following a Kruskal–Wallis test (*p* < 0.05) in both ESI (−) and ESI (+) modes of ionization

Previously, the relevance of the high levels of *PROSYS* in plant defence responses was described with respect to wild-type plants. The impact of SYS in plant metabolism has been studied through a stimulus that increased expression of the *PROSYS* gene [33, 37]. Thus, we wondered whether the increased levels of SYS in plant tissues could significantly alter plant metabolism. To study the level and relevance of the impact of SYS in the metabolome, a comparative metabolic analysis of shoots of wild-type, PS+ and PS− plants was conducted. An untargeted analysis was performed using UHPLC coupled to a Q-TOF (quadrupole-time of flight) mass spectrometer. The analysis was carried out in both positive and negative electrospray ionization (ESI) modes to cover a wider spectrum of compounds under the influence of SYS. Metabolic and bioinformatic analyses of the signals obtained were performed following the procedure described by Fernie et al. [38], Kaever et al. [39], and Xia et al. [40]. Score 3D plots of a PLS (Fig. 4a) showed three major components contributing to separation of the three groups. In both ionization modes, a clear separation of the three conditions was observed, with 76% of the total components contributing to the differences in ESI (−), while 92.6% of the components may explain the variance among groups. This experiment was performed in the absence of stress, so the observed phenomena can be attributed only to the constitutive levels of SYS in the three different genotypes. Following a partial least-square (PLS) regression analysis, it was observed that the absence of SYS in PS− showed relevant differences compared with the other two conditions (Fig. 4a; Additional file 5: Table S2). Although the three groups of samples were clearly separated, PS+ and BB plants remained closer, while PS− showed a very different metabolic behaviour. The differences between the three conditions were visualized through supervised heatmaps (Kruskal–Wallis test, *p *< 0.05). Figure 4b shows the clustering and heatmap of those compounds with higher differences among all signals extracted following statistical analysis. Interestingly, in the negative mode, PS+ exhibited a higher number of accumulated compounds, while PS− and BB clustered together. In contrast, on ESI (+), PS+ and PS− clustered closely. Nevertheless, the signals in PS+ were still significantly different with respect to the other two conditions, demonstrating a strong influence of the SYS peptide in plant metabolism despite the low changes found in PS+ overexpressing plants. This finding concurs with the consideration of SYS as a peptidic hormone. It has been demonstrated that the expression of the tomato *PROSYS* gene in other plant species also has a major impact on the proteomic profile [41], highlighting the impact that SYS produces in plants, either at the metabolic, proteomic, or transcriptomic levels [33]. Notably, all studies [33, 41] were performed in the absence of stress.

### SYS has a major impact on the lignan and tyrosine metabolic pathways

To elucidate the regulating/signalling function of the SYS peptide in plants, a study of the metabolic pathways was performed, considering the signals (metabolites) showing specific profiles of accumulation under the proposed conditions (BB, PS+ and PS−). One of the challenges in non-targeted metabolic analysis is to identify the signals showing relevant changes in intensity. Using MarVis 2.0 [42] software, those metabolites with homogeneous behaviour among different experimental conditions, including BB, PS+ and PS−, were grouped, assigned and associated with different metabolic pathways. Identification was performed at three different levels by a match in the exact mass, comparative fragmentation spectrum (Metlin (https://metlin.scripps.edu/), MassBank (http://massbank.jp), and the Kegg *Solanum lycopersicum* databases, for pathway assignments [43] and retention times of the fragments using internal libraries.

**Fig. 5:**
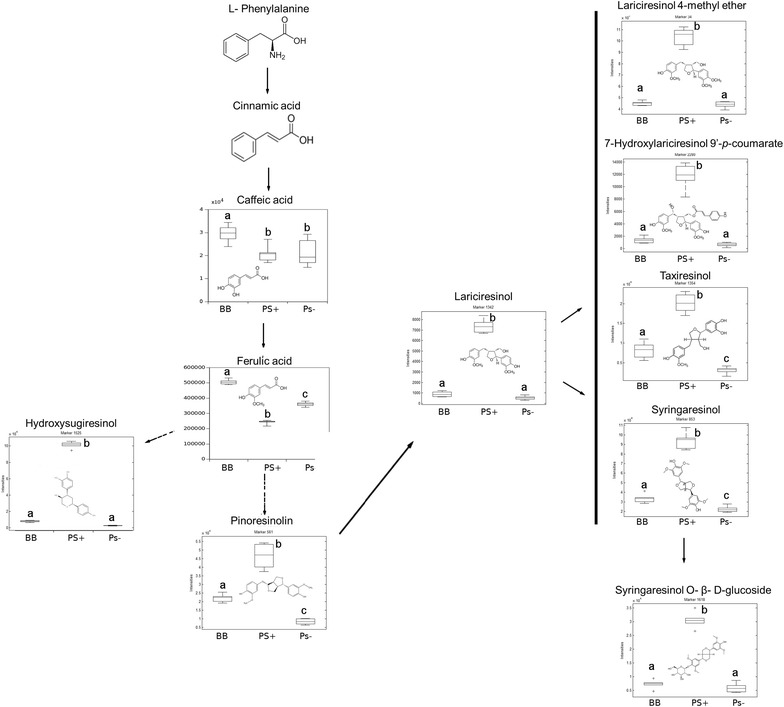
Profile of selected lignans and their precursor metabolites. 6-week-old plants were processed for relative quantification analysis in a UHPLC-QTOF MS. The metabolite concentration of each metabolite was normalized by using the chromatographic area for each compound with the dry weight of the corresponding sample. The third and fourth leaves of 3 individual plants 6-weeks-old was harvested and pooled for each genotype. Box plots represent the means for three independent experiments with two technical replicates. Different letters indicate statistically significant differences (ANOVA, Fisher’s least significant difference (LSD) test, *p *> 0.05, n = 6)

We then focused on signals showing higher accumulation in PS+ plants to identify systemin-regulated metabolic processes. Following MarVis Pathway analysis [42], PS+ plants showed a strong over-representation of metabolites within the lignan pathway (Additional file 4: Table S1). Lignans are an abundant class of phenylpropanoids defined as dehydrodimers of monolignols that are optically active and widely spread in the plant kingdom [44]. Monolignols are derived from phenylalanine (Phe) through several enzymatic reactions, including phenylalanine ammonia lyase (PAL) [25]. Endogenous SYS showed a major impact in the lignan and tyrosine pathways, among others (Table 2). Interestingly, the accumulation of lignans, stimulated by SYS, is preceded by a reduction in their precursor compounds, such as caffeic and ferulic acids, originating from the phenylpropanoid biosynthesis pathway (Fig. 5). Precursor compounds showed a decrease in PS+, , possibly leading to a higher synthesis of lignans in PS+ plants. Interestingly, some of these tentative lignans, such as pinoresinolin, taxiresinol and syringaresinol, were less accumulated in PS− plants, pointing out the relevance of the presence of SYS in the accumulation of these lignans. Similar profiles were observed for metabolites related to different pathways, such as tyrosine metabolism (Table 2; Additional file 5: Table S2) and were also found to be less represented in the antisense PS− plants (Additional file 5: Table S2). Tyrosine metabolism was detected in both, negative and positive ESI. This metabolic pathway might participate in the formation of lignans through the phenylpropanoid pathway, as described previously [25].

**Table 2:** Assignment of active metabolic pathways in PS+

ESI (−) 205 markers in 82 sets	ESI (+) 85 markers in 60 sets
Lignans Tyrosine metabolism ABC transporters Flavonoid biosynthesis	Porphyrin and chlorophyll metabolism Ubiquinone and terpenoid-quinone biosynthesis Tyrosin metabolism ABC transporters

Main metabolic pathways responsive to higher levels of SYS found in PS+ in both modes of ionization ESI (+) and ESI (−). MarVis Pathway package assigned the signals to metabolic pathways according to *Solanum lycopersicum* data base. Sets means all the pathways assigned by the software that can be implicated in systemin action

Another highly represented pathway in PS+ plants were the ABC transporters-substrates compound pathway (Table 2). These results suggest both an enhancement of the trafficking of metabolites in the plasma membrane and increased cellular communication, likely orchestrated by the peptide SYS [45, 46]. Among the pathways affected by endogenous SYS, several compounds were selected that match the profile of being much higher in PS+ plants but much lower in PS− plants than in the control plants. Exact mass or exact mass and fragmentation spectrum were used for the identification of these compounds (Additional file 5: Table S2). The use of MarVis for identification has some limitations; this is because we only consider the annotated compounds as tentatively identified, and a full identification using pure standards, when available, would be desirable to fully characterise the identity of compounds altered by SYS in plants.

All these results strongly suggest that endogenous SYS participates in the reorganization of specific cell wall components in tomato plants which, in turn, may participate in cell wall reinforcement. Among the identified lignans, there are some glycosylated forms, possibly storage forms of lignans that may accumulate in vacuoles. The mode of lignan transport is still a subject of active research. Interestingly, in our study, one of the relevant identified pathways comprised compounds participating in the ABC transporter pathway. Scranton et al. [47] have reported incremental accumulation of monolignol biosynthesis genes and flavonoids (anthocyanins) after injury in tomato leaves. The overexpression of *PROSYS* is described as the behaviour of a permanently injured plant, which releases PROSYS constitutively [19], and as shown in the present study, lignan and flavonoid biosynthesis are two of the pathways altered in PS+ plants.

**Fig. 6:**
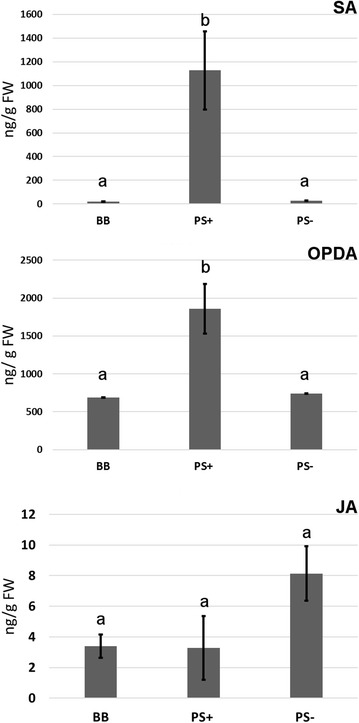
Basal levels of the main defense-related hormones in the wild-type, overexpressor and antisense PROSYS plants. Leaves from six-week-old wild-type, PS+ and PS− plants were harvested for SA, OPDA and JA quantification. Samples from six independent experiments were used for the analysis. Different letters indicate statistically significant differences (ANOVA, Fisher’s least significant difference (LSD) test, *p *> 0.05, n = 6)

Transcriptomic and proteomic analyses show that *PROSYS* overexpression has a considerable impact on the genes and proteins implicated in both oxidative stress and defence [33, 41], but there is no information regarding effects at a metabolomic level. Remarkably and unexpectedly, the overexpression of *PROSYS* was shown to induce SA-responsive genes and the jasmonic acid (JA) negative regulators *JAZ1* and *JAZ3* [33]. Our study corroborates this result at the metabolic level, where higher SA levels, but not JA levels, were found in PS+ plants (Fig. 6). This is an interesting finding, since the presence of PROSYS in plants has been associated with the enhancement of JA or its derivatives. In fact, PROSYS was shown to display enhanced *PI1* and *PI2* basal gene expression [19], and therefore, PS overexpressing lines have been used in multiple studies as JA overaccumulators. In our experiments, among the six independent biological replicates, we did not find more basal JA accumulation in PS+ plants; however, its precursor, OPDA, accumulated significantly. This observation calls for a reconsideration of PS+ as a JA overaccumulator.

To confirm whether the accumulation of lignans is a SYS-dependent effect, BB wild-type tomato plants were treated exogenously with the peptide SYS, and metabolomics analysis under the same conditions as described above was performed. SYS applied exogenously resulted in strong impacts on the lignin, ABC transporters-substrate compound, tyrosine metabolism and phenylpropanoid biosynthesis pathways in the SYS-treated plants compared with water-treated controls (Additional file 6: Table S3). This observation confirms and highlights a strong metabolomic overlap between SYS-treated and PS+ overexpressing plants. Further studies are needed to elucidate the exact relation between SYS and lignan metabolism.

## Conclusions

Peptides are attracting more and more attention due to their involvement in the defence processes, although detection and quantification are challenging issues, since peptide levels might be very low. Thus, the combination of very sensitive instrumentation and methods with reduced sample manipulation are desirable to get a precise and accurate quantification of the peptide of interest. PS+ plants accumulate endogenous SYS, leading to metabolic changes that may affect other physiological responses. The major changes observed in our study support an important regulatory role of SYS in the synthesis of metabolites implicated in cell wall structure. SYS quantification by an easy and simple method like ours allows the possibility of precisely exploring systemin regulation under stress conditions or upon plant elicitation, thereby establishing the role of this peptide in plant physiology.

## Methods

### Plant material and growth conditions

Wild-type tomato plants (*Solanum lycopersicum*), *BetterBoy* variety, and transgenic lines (overexpressor *35S::PROSYS* and antisense of PROSYS gene) lines were provided by the Ryan laboratory and were referenced in McGurl et al., 1992 and 1994 [18, 19]. All plants were grown in a growth chamber under 16 h of light (300 µE m^−2^ s^−1^) at 26 °C and 8 h of dark at 22 °C. The tomato seeds were sown in vermiculite, and when cotyledons were developed, the plantlets were transferred to 300 ml pots with vermiculite:soil (1:1) mixture and watered three times a week with Long Ashton solution [48]. After 6 weeks, the germinated samples were harvested and placed in − 80 °C until analysis.

### SYS treatment

Seeds of the wild-type plants of *BetterBoy* cultivar were sown in jiffy-7 pots. Plants were treated by soil drenching with 10 nM (as final concentration) of the peptide SYS (http://www.genscript.com/) 48 h before of harvesting. Leaves were harvested after 6 weeks and placed in − 80 °C until analysis.

### Reagents and standards

Supergradient HPLC-grade methanol and acetonitrile were purchased from Scharlab (ME 0306 and AC 0331, respectively). Formic acid was obtained from J.T. Baker (Deventer, Holland, 6037). Labelled and non-labelled peptides were purchased from Genscript. Lignan standards were purchased in Sigma.

### Liquid chromatography-tandem mass spectrometry (LC–MS/MS) for SYS analysis

High-performance liquid chromatography (HPLC) was performed by using a Waters Xevo TQ-S. Aliquots of 20 µl were injected into the system through a reversed column Aeris PEPTIDE 3.6 µ XB-C18 (150 × 4.6 mm) from Phenomenex, with a flow rate of 0.3 ml min^−1^. SYS was eluted with a flow of a gradient of ACN (organic phase) and Milli-Q water containing 0.1% of HCOOH (aqueous phase), starting with 5:95 (v/v), reaching 35:65 (v/v) linearly over 10 min and plateauing at 95:5 (v/v) 1 min later. The gradient was kept in isocratic conditions for 1 min before the column was left to equilibrate for 3 min in order to reach initial conditions, for a total of 15 min per sample.

The effluents originating from the HPLC were introduced into a triple quadrupole mass spectrometer (Xevo TQS, Waters Micromass, Manchester, UK) equipped with T-Wave devices and an ESI interface operated in positive mode. The cone and desolvation gas was nitrogen. The nebulizer gas flow was set to 250 L h^−1^ and the desolvation gas flow at 1200 L h^−1^. For operation in tandem MS/MS mode, the collision gas was pure 99.995% argon (Praxair, Madrid, Spain), with a pressure of 4 × 10^−3^ mbar in the collision cell. The desolvation gas temperature was 650 °C, the source temperature was set at 150 °C, and capillary voltage was 3.2 kV. The mass spectrometer was set in multiple reaction monitoring (MRM) mode and the data were acquired and processed using the MassLynx v 4.1 software (Waters, Manchester, UK).

### LC-ESI full scan mass spectrometry (Q-TOF instrument)

Freeze-dried leaves (30 mg per sample) were homogenized on ice in 1 ml of MeOH:H_2_O (10:90) containing 0.01% of HCOOH with metal balls (2 mm ø). The homogenates were centrifuged at 14000 rpm for 20 min at 5 °C. The supernatant was recovered and filtrated through 0.2 µm of regenerated cellulose filters (Teknokroma). An aliquot (20 µl) of the filtered extract was used for LC–MS analysis. Full metabolomics profiling was carried out by non-targeted analysis using an Acquity UPLC system (Waters, Milford, MA, USA) interfaced to a hybrid quadrupole time-of-flight mass spectrometer (Q-TOF MS Premier). The LC separation was performed with a Kinetex C18 analytical column, 1.7 µm particle size, 50 mm × 2.1 mm (Phenomenex). Elution of metabolites was performed using a gradient of methanol and water, both containing 0.01% of HCOOH. The gradient started with an aqueous solvent al 95% and a flow of 0.3 ml min^−1^. The gradient reached 50% of aqueous solvent at 8 min, increasing the level of organic solvent to 95% at 12 min. The gradient was kept in isocratic conditions for 1 min and later returned to initial conditions in 2 min. The column could equilibrate for 3 min, for a total of 22 min per sample. The library of compounds used for straight compound identification as well as the Q-TOF MS parameters were set as described by Gamir et al. (2014) [49].

### Liquid chromatography-tandem mass spectrometry (LC–MS/MS) for hormone analysis

Plant samples were stored at − 80 °C and approximately 350 mg of fresh material per sample was transferred into 2 ml microcentrifuge tubes. Ultrapure water (Millipore, www.merckmillipore.com) with a solution of internal standards was added to the microtubes (SA-d5 and dhJA (Sigma-Aldrich)). The contents of the tubes were homogenized with glass beads (2 mm ø), and extractions were performed in a mixer mill at a frequency of 30 Hz for 3 min. The tubes were centrifuged at 13.000 rpm for 30 min. Supernatants were adjusted to a pH of 2.5 with acetic acid 50% (v/v), and extractions were partitioned twice against diethyl ether. Organic fractions were concentrated to dryness in a centrifugal evaporator (Speedvac) at room temperature. The samples were resuspended in 1 ml of H_2_O/MeOH (90:10), leading to a 100-ng ml^−1^ final concentration of the internal standards. The columns used for chromatographic separation were the same as described above, and the chromatographic conditions and TQD parameters used were described by Gamir et al. (2014) [49].

### Full-scan data analysis

Raw data were transformed to.cdf files using the DataBridge package provided by the Masslynx 4.1. Signals derived from ESI (+) and ESI (−) were processed separately. Peak peaking, grouping and signalling corrections were processed with R software for statistical computing using the XCMS package for relative quantification [50]. Partial least-squares (PLS), heatmap construction and clustering were performed with MetaboAnalyst 3.0 [40]. The Kruskal–Wallis test (*p *< 0.05) was applied to show differences between conditions. For signal identification, MarVis Pathway software was used [42]. Syringaresinol O-beta-d-glucoside, taxiresinol, epysyringaresinol, lariciresinol, and pinoresinol standards were used as an internal library for full identification.

### RNA extraction and RT-qPCR analysis

Measurements in gene expression were performed by quantitative RT-qPCR using RNA samples extracted from third and fourth true leaves. The process of RNA extraction was adapted from Valledor et al. [51]. Samples were treated with DNAse I (Takara) following the manufacturer’ instructions. To obtain cDNA, 1.5 µg of RNA was annealed to oligo-dTs, and retrotranscription was performed with a PrimeScript RT reagent kit (Perfect real-time) from Takara. RT-qPCR was conducted using Maxima SYBR Green/ROX qPCR (Thermo Scientific) on a StepOne instrument (Applied Biosystems). For optimum amplification efficiency, a standard curve through serial dilutions of cDNA was constructed. Specificity of RT-qPCR amplification was tested by looking for a single peak in the melting temperature curve analysis. Relative quantification of mRNA levels was calculated using the comparative $2^{- \Delta \Delta C}_{\text{T}}$ method [52]. For normalization of expression values, two housekeeping genes were used: the tomato elongation factor *EF*-*1* (*fw*- 5′-GATTGGTGGTATTGGAACTGTC-3′; *rev*- 5′-AGCTTCGTGGTGCATCTC-3′) and the tomato *ACT*-*52* gene (*fw*- 5′-CACCATTGGGTCTGAGCG-3′; *rev*- 5′-GGGCGACAACCTTGATCT-3′). Relative expression data between the studied gene *PROSYS* (*fw*- 5′-AATTTGTCT CCCGTTAGA-3′; *rev*- 5′-AGCCAAAAGAAAGGAAGCAAT-3′) and the housekeeping genes were calculated from the differences in threshold cycle (∆Ct).

### Statistical analyses

All statistical analyses were conducted with Statgraphics Centurion software for the ANOVA, with post hoc and *t test* analysis. The xcms package in the R software version 3.1.2 was used for these analyses. All experiments were repeated at least three times, unless otherwise noted.

## Additional files

**Additional file 1: Figure S1.** Positive precursor ion and MS/MS product ion mass spectra for SYS. SYS standards were infused into the MS analyser at the concentration of 1 mg l^−1^.(a) Parental ions for SYS; (b) spectrum fragmentation of [SYS + 4H]^4+^.

**Additional file 2: Figure S2.** Optimized fragmentation spectrum of SYS and SYS*. The product ions obtained by ESI (+) corresponding to 503.3 and 505.1 were fragmented to obtain the fragmentation spectra for SYS and SYS* and optimized for the transitions to 143.1 and 148.1 respectively.

**Additional file 3: Figure S3.** HPLC–MS/MS chromatograms of labelled SYS. Chromatograms obtained with 100 ng ml^−1^ of pure labelled SYS* dissolved in H_2_O: ACN (9:1) v/v in the same chromatographic conditions used for SYS standard.

**Additional file 4: Table S1.** Tentative identification of lignans accumulated in PS+ . Tentative candidates of lignans attributed to the endogenous SYS present in PS+ with their respective product ions in ESI (−). Identification and pathway assignments were done using the MarVis 2.0 software and an internal library, referred-to level 3 [43].

**Additional file 5: Table S2.** Relevant pathways and compounds under SYS control. List of compounds that show higher level intensities in PS+ , and lower ones in PS− respect to BB and PS+ . All the masses presented in the table have been annotated at a tentative level, although in some of them a further identification by using fragmentation spectrum has been performed. Abbreviations: fs, identification by exact mass and fragmentation spectrum level 2 of identification [43]; ti, is a tentative identification of the compound referred-to level 3 [43].

**Additional file 6: Table S3.** Tentative identification of lignans accumulated by exogenous SYS. Tentative candidates for lignans accumulated after two days of SYS application at 10 nM of final concentration applied in roots, in ESI (−). Identification and pathway assignments were done using the MarVis 2.0 software and an internal library (level 3 of identification [43]).

## Abbreviations

PROSYS: Prosystemin peptide
SYS: Systemin peptide
PS+: Overexpressor of Prosystemin
PS−: Antisense mutant of Prosystemin
MAMP: Microbe associated molecular pattern
DAMP: Damage associated molecular pattern
PTI: Pathogen Associated Molecular Pattern-triggered Immunity
AtPeps: Peptides in Arabidopsis
PEPR1 and PEPR2: Arabidopsis peptide receptor PEP- RECEPTOR 1
ZmPep: Peptides in *Zea mays*
PI: Proteinase inhibitor
HypSys: Hydroxyproline-rich systemins
MRM: Multiple reaction monitoring
ESI (+): Electrospray ionization in positive mode
ESI (−): Electrospray ionization in negative mode
