# Supplementary figures and images for: Accurate and easy method for systemin quantification and examining metabolic changes under different endogenous levels
Source: Plant Methods. 2018 Apr 26;14:33. doi: 10.1186/s13007-018-0301-z (PMC5918566; doi:10.1186/s13007-018-0301-z)

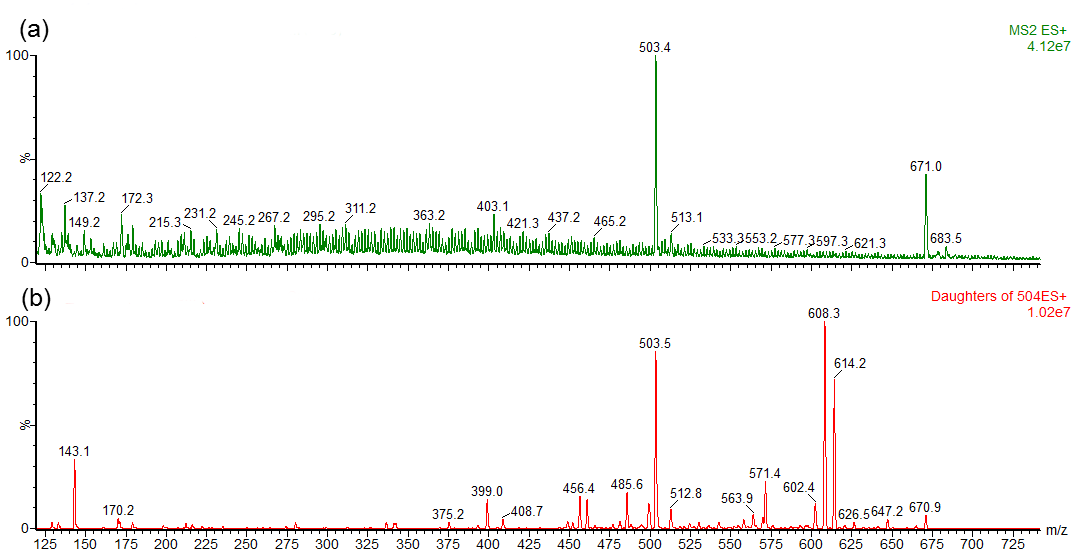

Supplement: Supplementary file 1 — Additional file 1: Figure S1. Positive precursor ion and MS/MS product ion mass spectra for SYS. SYS standards were infused into the MS analyser at the concentration of 1 mg l−1.(a) Parental ions for SYS; (b) spectrum fragmentation of [SYS + 4H]4+. [file 13007_2018_301_MOESM1_ESM.tif]

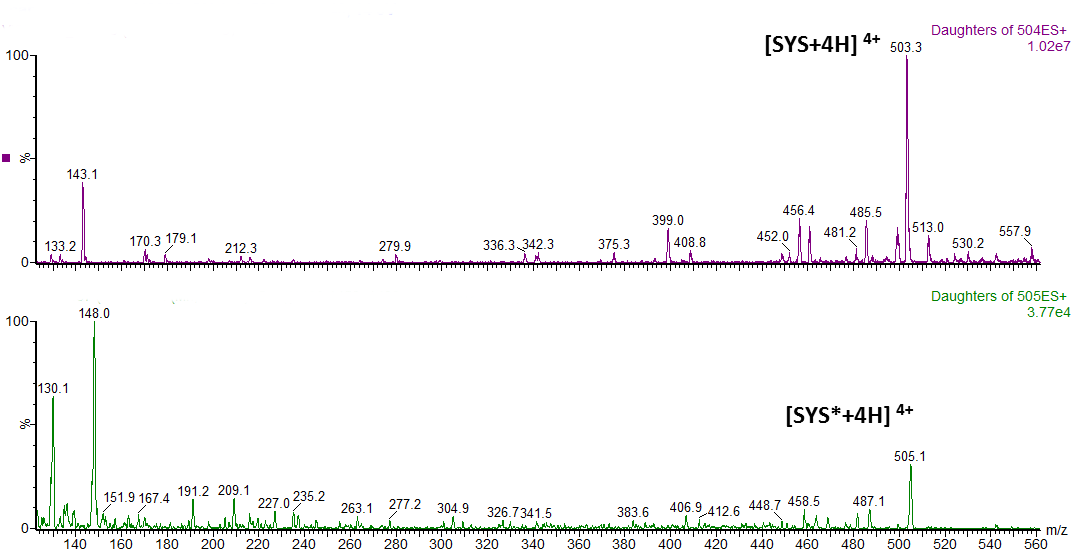

Supplement: Supplementary file 2 — Additional file 2: Figure S2. Optimized fragmentation spectrum of SYS and SYS*. The product ions obtained by ESI (+) corresponding to 503.3 and 505.1 were fragmented to obtain the fragmentation spectra for SYS and SYS* and optimized for the transitions to 143.1 and 148.1 respectively. [file 13007_2018_301_MOESM2_ESM.tif]

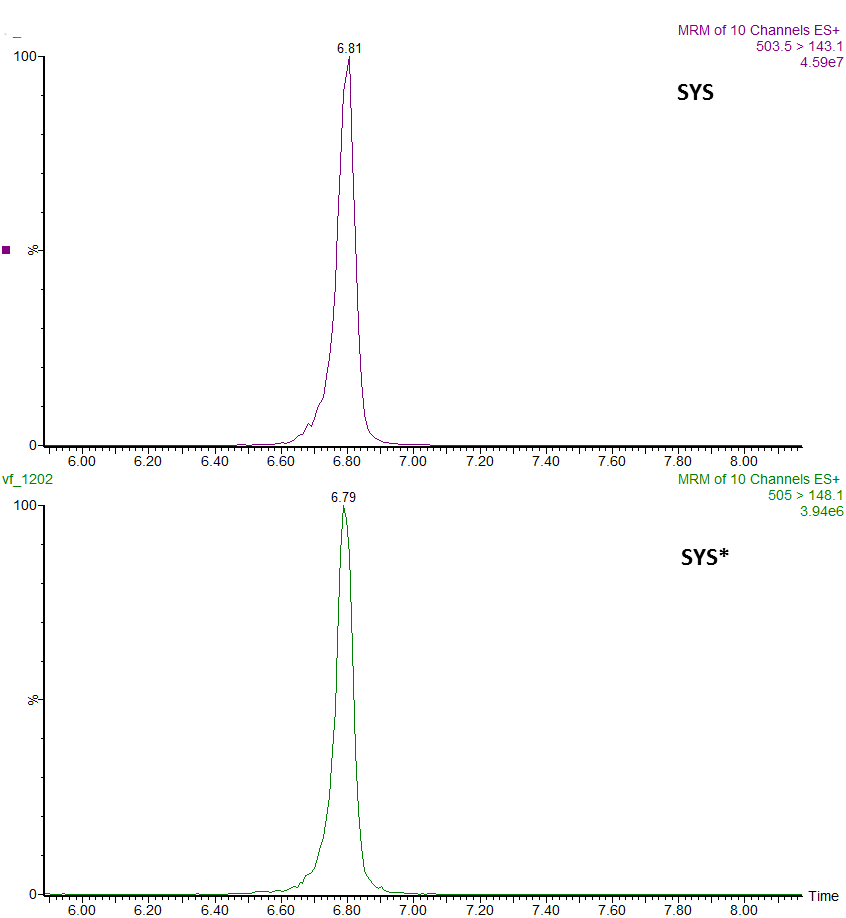

Supplement: Supplementary file 3 — Additional file 3: Figure S3. HPLC–MS/MS chromatograms of labelled SYS. Chromatograms obtained with 100 ng ml−1 of pure labelled SYS* dissolved in H2O: ACN (9:1) v/v in the same chromatographic conditions used for SYS standard. [file 13007_2018_301_MOESM3_ESM.tif]
