# Supplementary material for: Accurate and easy method for systemin quantification and examining metabolic changes under different endogenous levels
Source: Plant Methods. 2018 Apr 26;14:33. doi: 10.1186/s13007-018-0301-z (PMC5918566; doi:10.1186/s13007-018-0301-z)
Supplement: Supplementary file 4 — Additional file 4: Table S1. Tentative identification of lignans accumulated in PS+ . Tentative candidates of lignans attributed to the endogenous SYS present in PS+ with their respective product ions in ESI (−). Identification and pathway assignments were done using the MarVis 2.0 software and an internal library, referred-to level 3 [43]. [file 13007_2018_301_MOESM4_ESM.docx]

**Additional file 4: Table S1.** **Tentative identification of lignans accumulated in PS+.** Tentative candidates of lignans attributed to the endogenous SYS present in PS+ with their respective product ions in ESI (-). Identification and pathway assignments were done using the MarVis 2.0 software and an internal library, referred-to level 3 [43].

| **LIGNANS** | **[M-H^-^ ]** |
| --- | --- |
| Hydroxysugiresinol | 301.0769 |
| 7-Hydroxylariciresinol 9’-p-coumarate | 521.1422 |
| Syringaresinol O-beta-D-glucoside | 579.2281 |
| Lyoniresinol 3alpha –O-beta-glucopyranoside | 581.1788 |
| Syringaresinol | 417.1779 |
| Taxiresinol | 345.1573 |
| Pinoresinolin | 373.1156 |
| Episyringaresinol | 417.1779 |
| Pinoresinol –diglucoside | 682.2446 |
| Zhepiresinol | 279.0929 |
| Lariciresinol 4-methyl ether | 373.1881 |
| Lariciresinol | 359.0960 |
