# Supplementary material for: Accurate and easy method for systemin quantification and examining metabolic changes under different endogenous levels
Source: Plant Methods. 2018 Apr 26;14:33. doi: 10.1186/s13007-018-0301-z (PMC5918566; doi:10.1186/s13007-018-0301-z)
Supplement: Supplementary file 6 — Additional file 6: Table S3. Tentative identification of lignans accumulated by exogenous SYS. Tentative candidates for lignans accumulated after two days of SYS application at 10 nM of final concentration applied in roots, in ESI (−). Identification and pathway assignments were done using the MarVis 2.0 software and an internal library (level 3 of identification [43]). [file 13007_2018_301_MOESM6_ESM.docx]

**Additional file 6: Table S3. Tentative identification of lignans accumulated by exogenous SYS.** Tentative candidates for lignans accumulated after two days of SYS application at 10 nM of final concentration applied in roots, in ESI (-). Identification and pathway assignments were done using the MarVis 2.0 software and an internal library (level 3 of identification [43]).

| LIGNANS | [M-H^-^ ] |
| --- | --- |
| Isolariciresinol 4-O-beta-D-glucopyranoside | 523.1728 |
| Piperitol | 355.1307 |
| Syringaresinol O-beta-D-glucoside | 579.2281 |
| Lyoniresinol 3alpha –O-beta-glucopyranoside | 581.1788 |
| Zhepiresinol | 279.0929 |
| 1-Hydroxypinoresinol 1-glucoside | 535.1959 |
